# Skull base approaches in neurosurgery

**DOI:** 10.1186/1758-3284-2-16

**Published:** 2010-07-05

**Authors:** Martin Scholz, Richard Parvin, Jost Thissen, Catharina Löhnert, Albrecht Harders, Klaus Blaeser

**Affiliations:** 1 Department of Neurosurgery, Klinikum Duisburg, Academic Teaching Hospital of University Essen-Duisburg, Germany; 2 Ruhr-University Bochum, Germany; 3 Department of Neurosurgery, Knappschaftskrankenhaus, Germany

## Abstract

The skull base surgery is one of the most demanding surgeries. There are different structures that can be injured easily, by operating in the skull base. It is very important for the neurosurgeon to choose the right approach in order to reach the lesion without harming the other intact structures. Due to the pioneering work of Cushing, Hirsch, Yasargil, Krause, Dandy and other dedicated neurosurgeons, it is possible to address the tumor and other lesions in the anterior, the mid-line and the posterior cranial base. With the transsphenoidal, the frontolateral, the pterional and the lateral suboccipital approach nearly every region of the skull base is exposable.

In the current state many different skull base approaches are described for various neurosurgical diseases during the last 20 years. The selection of an approach may differ from country to country, e.g., in the United States orbitozygomaticotomy for special lesions of the anterior skull base or petrosectomy for clivus meningiomas, are found more frequently than in Europe.

The reason for writing the review was the question: Are there keyhole approaches with which someone can deal with a vast variety of lesions in the neurosurgical field?

In my opinion the different surgical approaches mentioned above cover almost 95% of all skull base tumors and lesions. In the following text these approaches will be described.

These approaches are:

1) pterional approach

2) frontolateral approach

3) transsphenoidal approach

4) suboccipital lateral approach

These approaches can be extended and combined with each other. In the following we want to enhance this philosophy.

## Ad 1) pterional approach

### Historical overview

In the 1970 s Yasargil [1,2] laid to the foundation of the pterional approach. The pterional approach allows the surgeon to address the circle of Willis and also pathological changes in the cavernous sinus area. In the 1960s Parkinson [3,4] championed a route that exposed the cavernous sinus with less retraction of the brain then earlier approaches. The pterional approach is the standard surgical treatment for aneurysms in the anterior circle of Willis. During the Cushing era the term "nole mi tangere" ("don't touch me") was a rule for neurological surgeons, which considered treating intracranial aneurysms. The forefathers of aneurysm surgery were Norman Dott [5] and Walter Dandy [6]. Dott reported the first surgical treatment of an intracranial aneurysm. His patient suffered from an aneurismal subarachnoid hemorrhage. Dandy [7] was the first to begin clipping aneurysms of the internal carotid artery. Tönnis [8] was one of the first to treat aneurysms in the circle of Willis. While Tönnis' route through the anterior part of the corpus callosum showed successful results, his first try to expose the aneurysm of the anterior communication artery by a subfrontal approach did not succeed.

### Surgical technique

**Figure 1:**
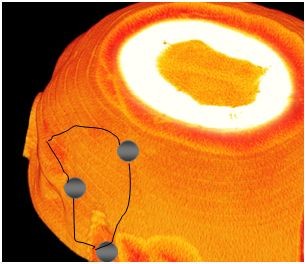
**Pterional approach on the left side**. Normally three burr holes are used.

**Figure 2:**
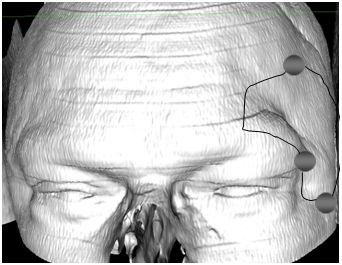
**Pterional approach from the frontal view**.

There are many variants for the pterional approach. Mainly it is a trepanation which permits access to the frontal and to the temporal lobe as well as the Sylvian fissure (Figure 1, Figure 2). After a curved skin incision the Galea is retracted. For treatment of the temporal muscle there are different variants which have all there clear indication. Many surgeons cut the muscle directly during skin incision and separating the combined musculocutaneous flap down until they reach pterion and the frontobasal are above the eye brow. The temporal muscle has to be separated carefully to approach the temporal bone.

Yasargil also described a technique that allows access to the same area: his skin incision is done until the temporal fascia is reached. He then creates a triangular galea flap and seperates the muscle after additional horizontal incision in order to protect the frontal branch of the facial Nerve. In our opinion the rate of nerve injury is minimal also if one is using the first technique. The advantage of the Yasargil Technique can be seen if one wants to combine the pterional approach with the subfrontal approach. The muscle can be retracted much easier downwards with this technique. In my opinion the rate of atrophic changes of the temporal muscle postoperatively is a little bit higher. We are using Palacaos to fill the burr holes. Other authors are using selected bone dust.

### Case report 1

A 51-year-old man underwent an operation for a craniopharyngioma, through a pterional approach from the right side six months before. He was presented with a pituitary infarction. He has been treated with Testosterone (Testogel), Desmopressin (Minirin) and Hydrocortisone. He suffered from a progressive visual disturbance (visual acuity r 0.6, l 0.8) for 8 weeks. A bitemporal hemianopsia was diagnosed.

### Outcome

**Figure 3:**
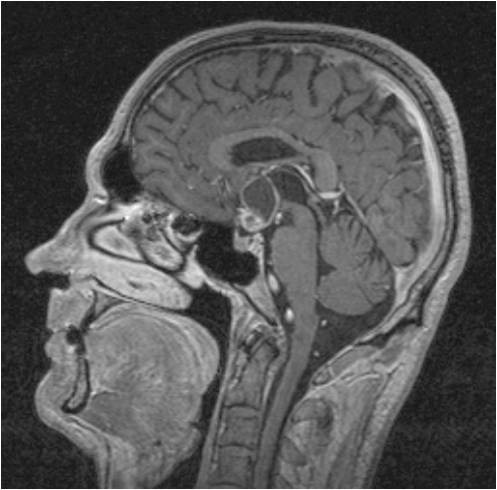
**Sagittal brain MRI of a 51 year old male with a recurrence of a craniopharyngioma**. The MRI demonstrates the hypertensive solid part and above, it demonstrates the cystic part.

**Figure 4:**
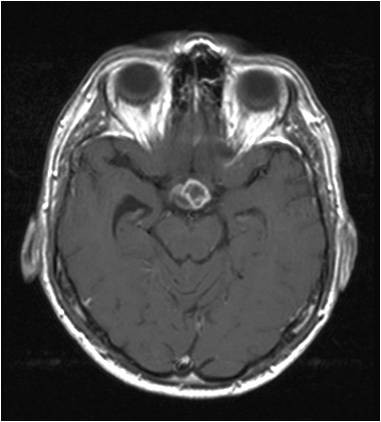
**Axial brain MRI of a 51 year old male**.

**Figure 5:**
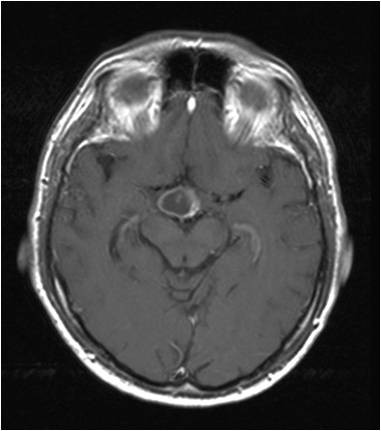
**Axial brain MRI of a 51 year old male in a different slice**.

We diagnosed a recurrence of the craniopharyngioma (Figure 3, Figure 4, Figure 5). Besides the postoperative right-sided temporal disturbance of the visual acuity, there were no other neurological deficits. The patient still has to substitute the endocrine system.

### Case report 2

**Figure 6:**
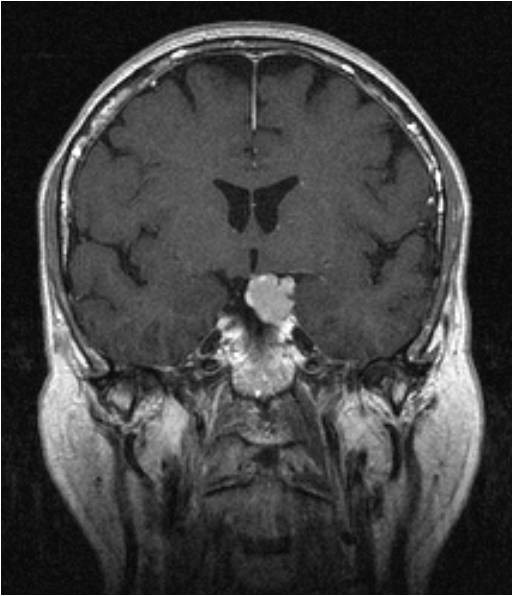
**MRI, T1 weighted images after administration of gadolinium, coronal section, demonstrates the circumscribed skull base tumor**.

**Figure 7:**
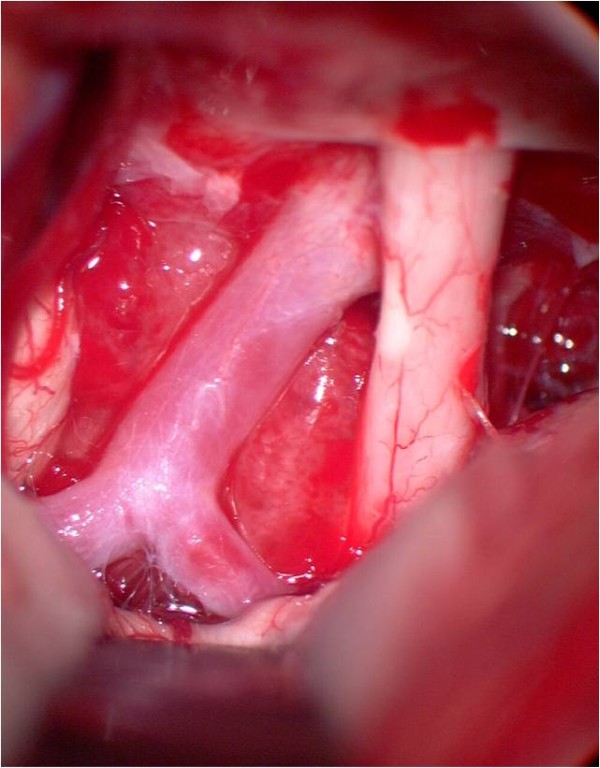
**Intraoperative view, left pterional approach**. The tumor bulges behind the internal carotid artery. In front of the tumor the left optic nerve can be seen. Behind the tumor the oculomotor nerve is demonstrated.

**Figure 8:**
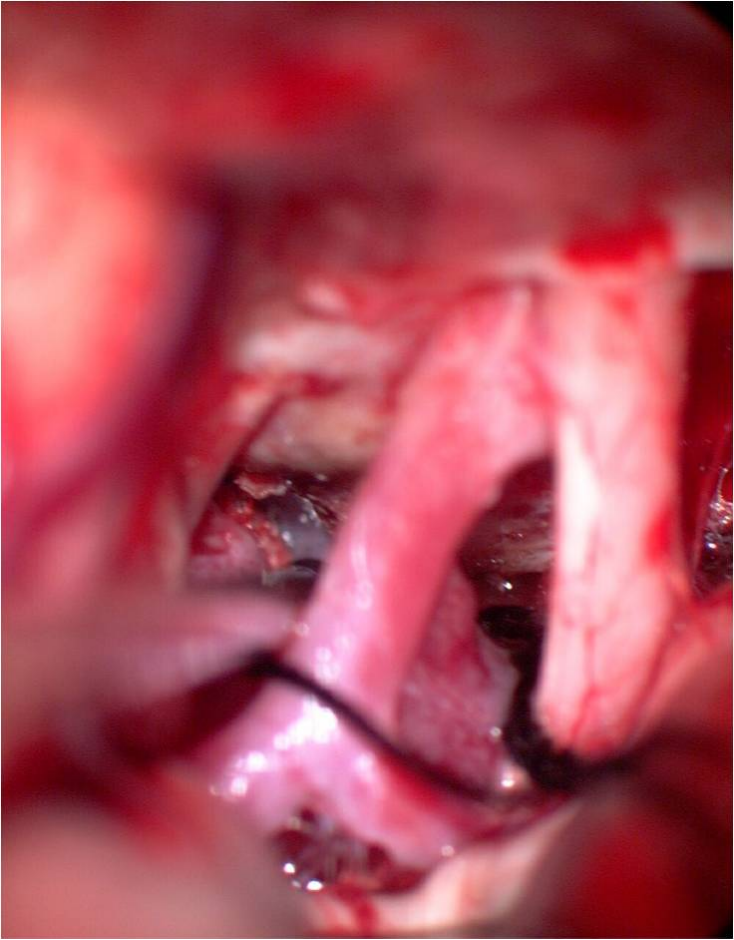
**After tumor removal**. The suction tip is located between internal carotid artery and oculomotor nerve.

**Figure 9:**
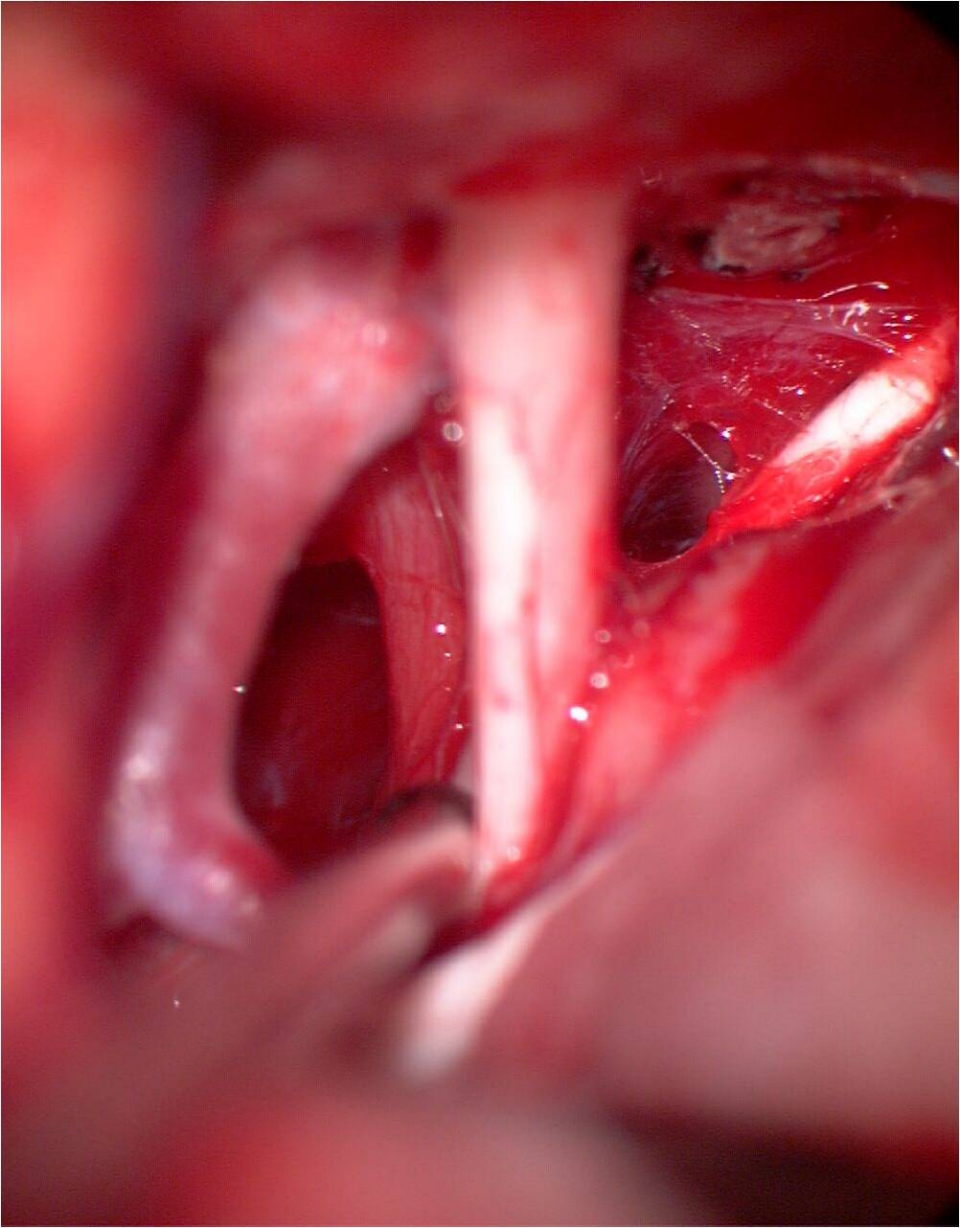
**Image of the pituitary stalk**. The pituitary stalk is demonstrated with the suction tip behind the chiasm.

64-year-old woman presented with unknown symptoms of vertigo and diplopia. Further there were no clinical deficits. The MRI showed a left space-consuming lesion latero para- and retrosellar of approximately 2 × 2,5 cm size (Figure 6). We suspected a dorsum sellae meningioma. The intraoperative view demonstrates the exact location of the tumor (Figure 7, Figure 8, Figure 9).

### Outcome

There were normal findings post-operatively with occasional diplopia, while the wound was healing per primam. Pathology revealed a meningothelial tumor of WHO grade I.

### Case report 3

66-year-old patient, presented with a history of six seizures, suffers from a known retrochiasmal lesion with a growth in the MRI examinations.

**Figure 10:**
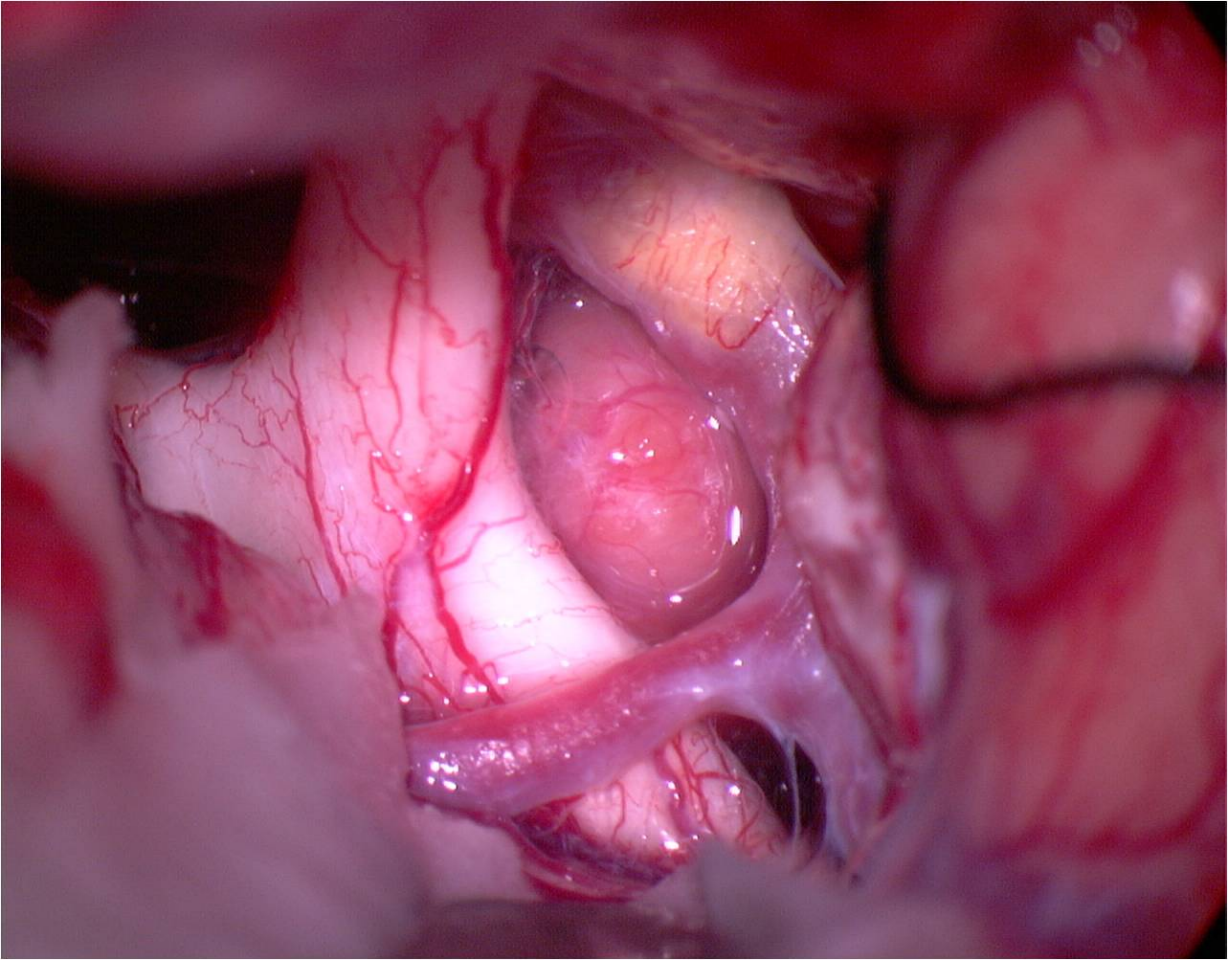
**Pterional approach**. Different steps during resection of a granular cell tumor of the pituitary stalk from the right side.

**Figure 11:**
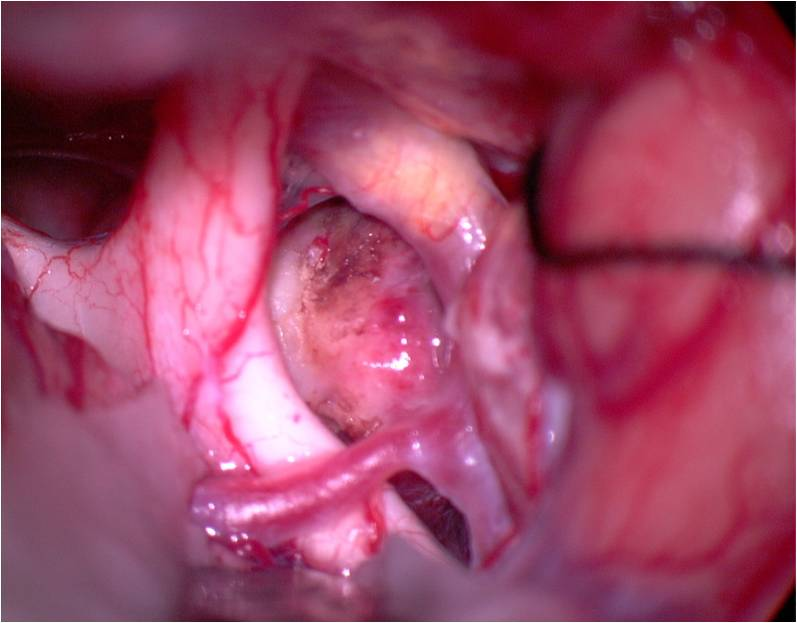
**Beginning of the resection via the pterional approach**. The chiasm is clearly visible.

**Figure 12:**
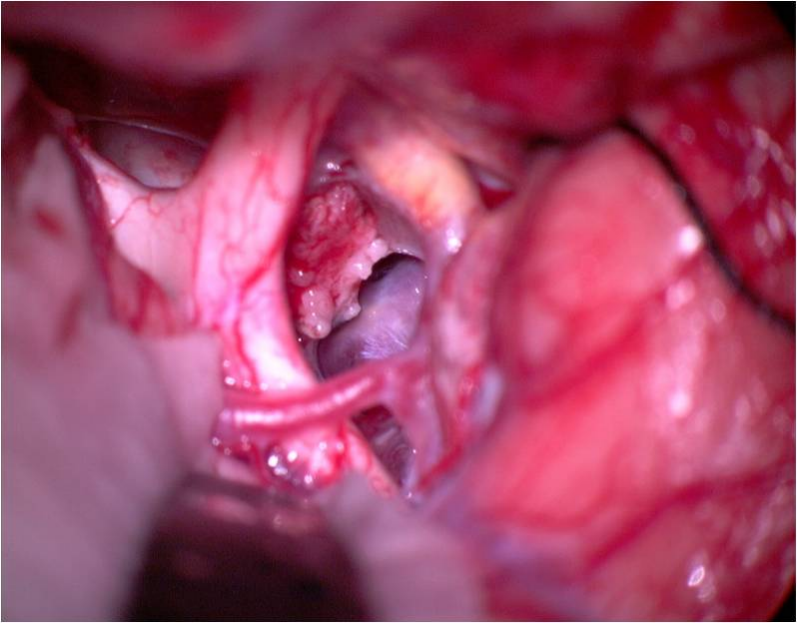
**After the resection of the tumor via the pterional approach**. Remnants of the tumor attached to the pituitary stalk are left behind.

We addressed the tumor via the pterional approach from the right side (Figure 10). The chiasm is visible during the resection (Figure 11). We used a grasping forceps and the sucker to shrink the tumor in a piecemeal fashion. A small residue was left around the infundibular stalk (Figure 12).

### Outcome

There were no neurological deficits and the healing of the wound was by first intention. In contrast the patient showed fluctuating vigilambulism with nausea, vertigo and electrolyte imbalance. The patient was prescribed a long-term hydrocortisone substitution and Desmopressin (Minirin) for a short period of time.

## Ad 2) frontolateral approach

### Historical overview

The frontolateral or unilateral subfrontal approach provides exposure of the anterior cranial base. It allows addressing olfactory groove meningiomas (OGMs) by a minimally invasive procedure and it is also one of the traditionally used approaches on OGMs [9,10]. Francesco Durante [11] marks the beginning of OGM extirpation. It was not until Fedor Krause [12], who used the unilateral subfrontal approach the first time to expose the sellar region. In contrast to Krause's tremendous impact on anterior fossa approach, Durante removed an OGM in 1885 via a transpalatine approach through an extension into the nasal fossae.

### Surgical technique

**Figure 13:**
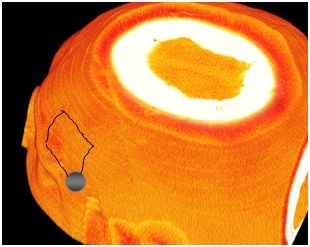
**Computer animation of a left frontolateral approach from the lateral view**. This trepanation can usually be done via one single burr hole.

**Figure 14:**
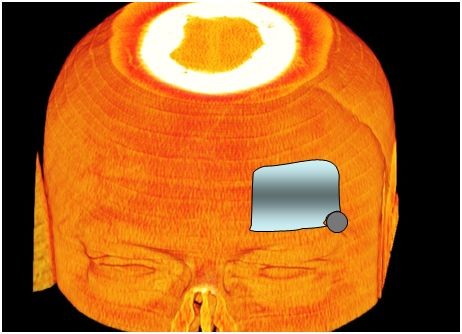
**Computer animation of a left frontolateral approach from the frontal view**. If the frontal sinus will be opened it has to be closed carefully with a galea flap.

This approach allows different skin incisions, which depend on the patient's anatomy and physiognomy. The osteoplastic trepanation above the pterion and above the temporal muscle follows a curved skin incision or an eyebrow skin incision [13] (Figure 13, Figure 14). The trepanation of an approximately 3 × 4-cm frontolateral craniotomy allows the entry to the anterior fossa. It is essential to include radiological data of the patient in order to prepare a performance of a sophisticated approach such as the frontolateral approach. A common iatrogenic injury is the injury of superficial structures. The superciliary skin incision allows the surgeon to protect superficial structures like the frontal branches of the facial nerve and the superficial temporal artery. Perneczky led to the development of endoscopical approaches using the supraorbital "key-hole" approach [14]. Via endoscopic techniques it is possible to provide relatively great exposure, while offering less brain retraction.

### Case report 4

A 47-year-old patient with bitemporal hemianopsia and a 6-weeks history of progredient headache was presented. The patient manifested no further neurological deficits.

**Figure 15:**
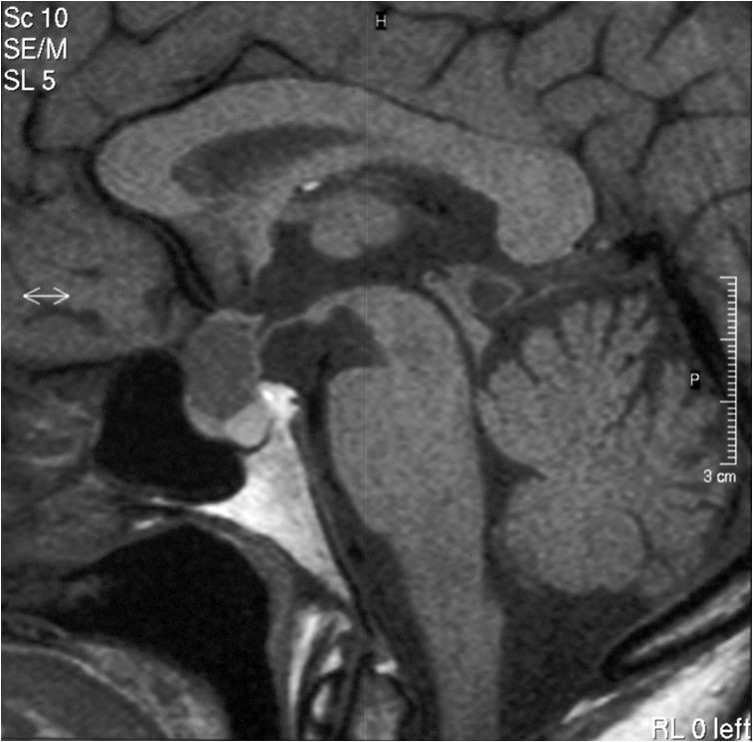
**MRI, T1 weighted images, saggital section**. A cystic lesion can be seen above the pituitary gland.

**Figure 16:**
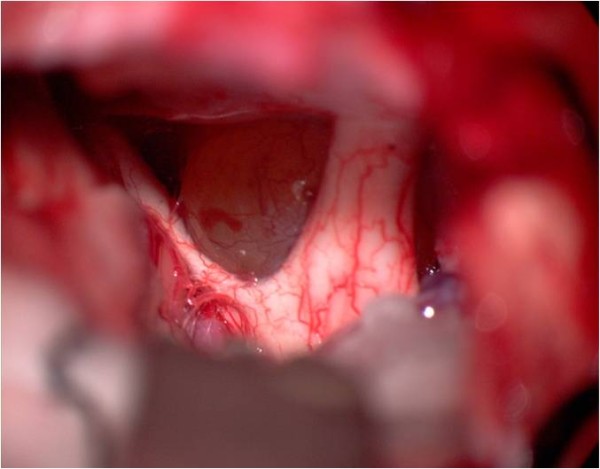
**Intraoperative view from the right side**. The cystic lesion (Rathke's cleft cyst) can be detected behind the optic nerves.

**Figure 17:**
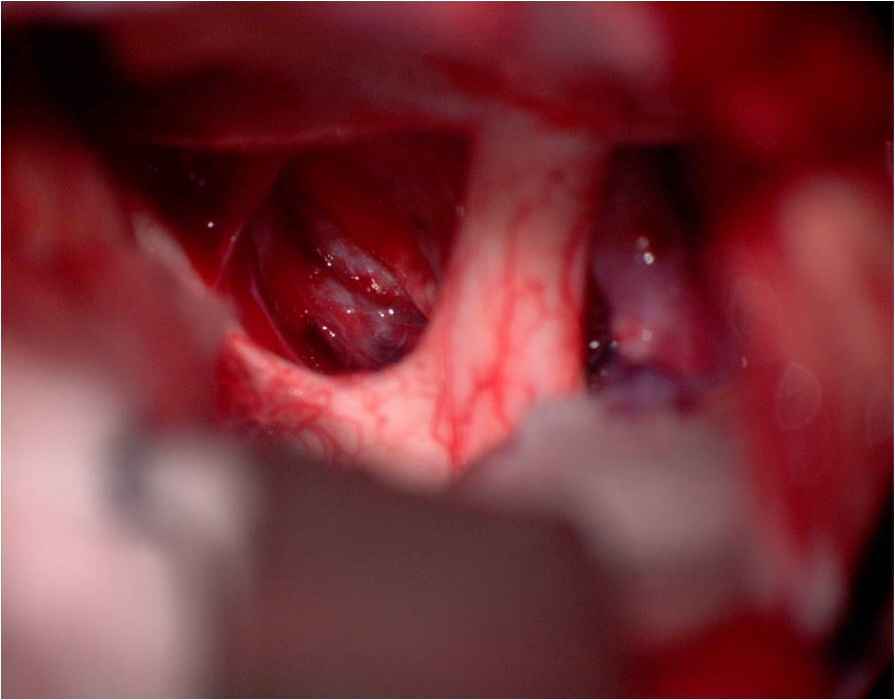
**After resection**.

Neuropathological findings revealed parts of an epithlial cyst inflammatory cell infiltration in connective tissue stroma. The MRI showed a cystic lesion above the pituitary gland (Figure 15). We performed a frontolateral approach, in order to expose the Rathke's cleft cyst (Figure 16) and to remove it (Figure 17).

### Outcome

The further course was without any complications and the wound healing was per primam.

Visual field defects were on the decrease. Furthermore, we couldn't recognize any hormonal failure.

## Ad3) transsphenoidal approach

### Historical overview

The transsphenoidal route was first used by the Egyptians in order to remove the brain. The pioneering work of Harvey Cushing [15] and Oskar Hirsch [16] led to one of the most efficient ways to operate in the sellar region. Cushing advocated the approach sublabially and Hirsch accessed the sellar region endonasally. Before Cushing and Hirsch led to the milestone in pituitary gland surgery, Sir Victor Horsley [17] was the first to operate on the pituitary gland. While the minimally invasive surgery trend is set in motion nowadays, the contemporary neurosurgeons would call Sir Horsley's approach too radical. His route required the retraction of the frontal lobe. In 1898 D. Giordano [18] developed the fundamental concepts to expose the pituitary gland via a transnasal-frontoethmoidal approach. But the contemporary standard route to the pituitary gland is carried out through the transsphenoidal approach.

### Surgical technique

**Figure 18:**
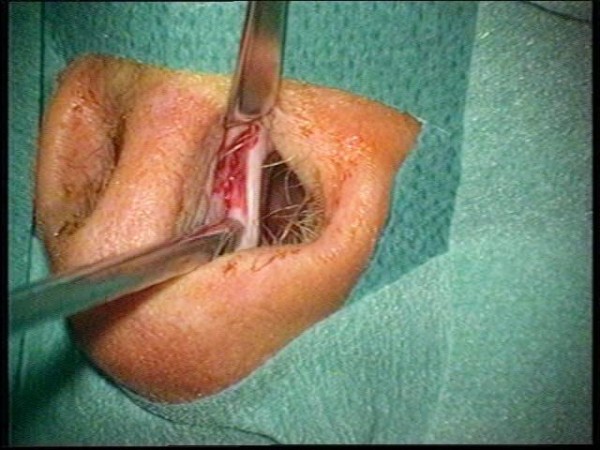
**Incision inside the nasal ostium**.

**Figure 19:**
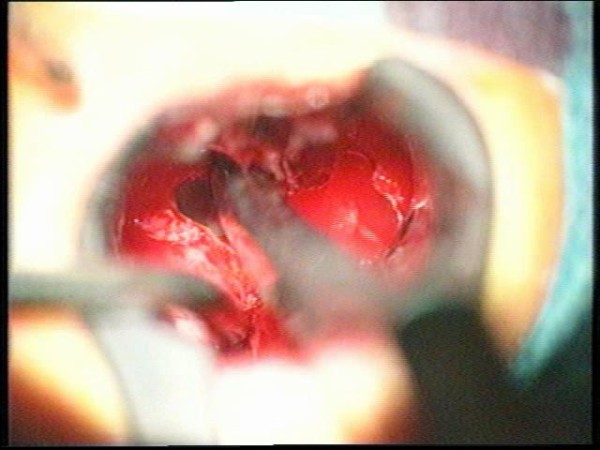
**Partial resection of bony septum**.

**Figure 20:**
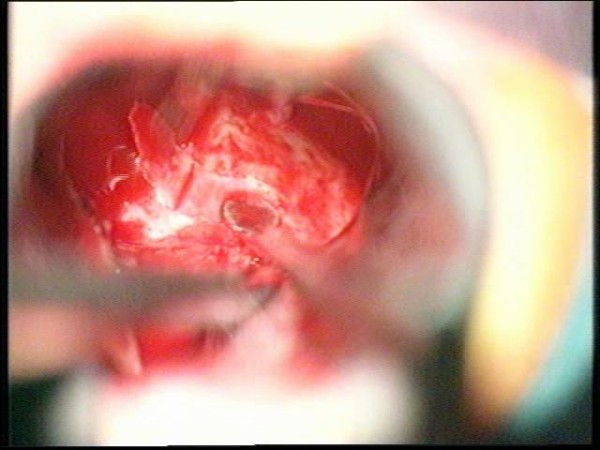
**Opening of the sellar floor**.

**Figure 21:**
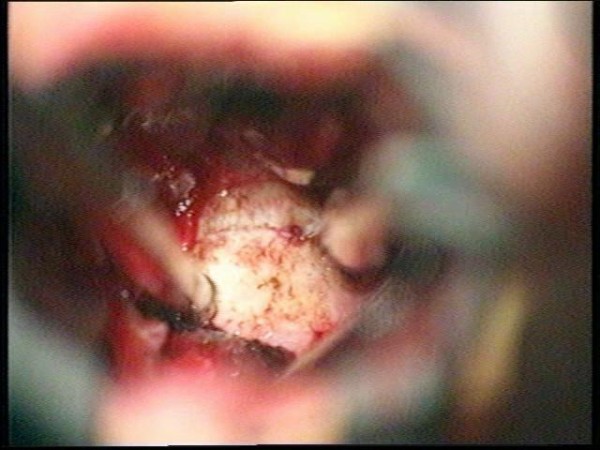
**The dura is exposed and opened**.

**Figure 22:**
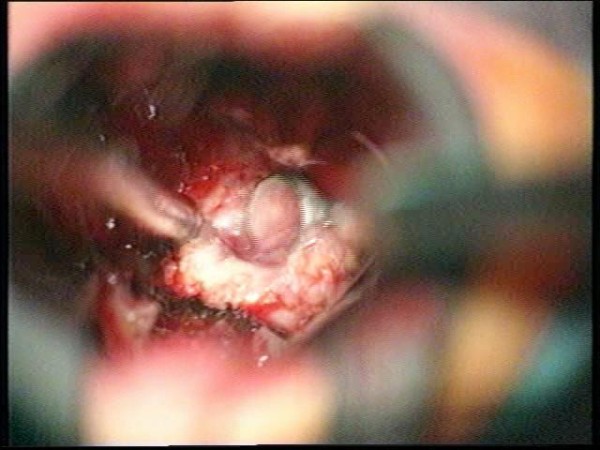
**The tumor is resected with a curette**.

The classic technique begins with the patient in supine position. An x-ray machine is positioned laterally to control each step of intervention. In microsurgery the surgeon stands at the tip of the head whereas in pure endoscopic pituitary surgery the neurosurgeon stands at the shoulder of the patient. We would like to concentrate on the microsurgical approach in our review article. After incising the septal mucosa (Figure 18) and revealing the anterior wall of the sphenoid sinus, the anterior wall of the sphenoid sinus will be removed with punch forceps (Figure 19, 20). We then excise the sinus mucosa. After opening the dura (Figure 21), the tumor must be indentified in order to extirpate it with a curette (Figure 22) by using lateral extensions. The transsphenoidal route is portrayed as the fundamental treatment of pituitary adenomas and other tumors in the sellar region [19,20].

## Ad 4) suboccipital lateral approach

### Historical overview

The posterior cranial base can be explored through the lateral suboccipital route. Processes in the posterior fossa such as acoustic neurinoma surgery can be carried out through the lateral suboccipital approach. The unilateral suboccipital approach was popularized by Woolsey (1903) and with great contributions by Krause (1905) [21,22]. After several refinements and modifications through different dedicated neurosurgeons (Fish [23], House [24,25] and Seiffert [26]), Dandy's [27] suboccipital approach (1917) with an ipsilateral suboccipital flap evolved to what we call retrosigmoid transmeatal approach now. Cushing (1917) [28] on the other hand described a bilateral suboccipital access and stated the unilateral suboccipital approach as disadvantageous. In modern neurosurgery Majid Samii [29,30] is considered to be one of the leading experts in vestibular nerve schwannoma surgery.

### Surgical technique

**Figure 23:**
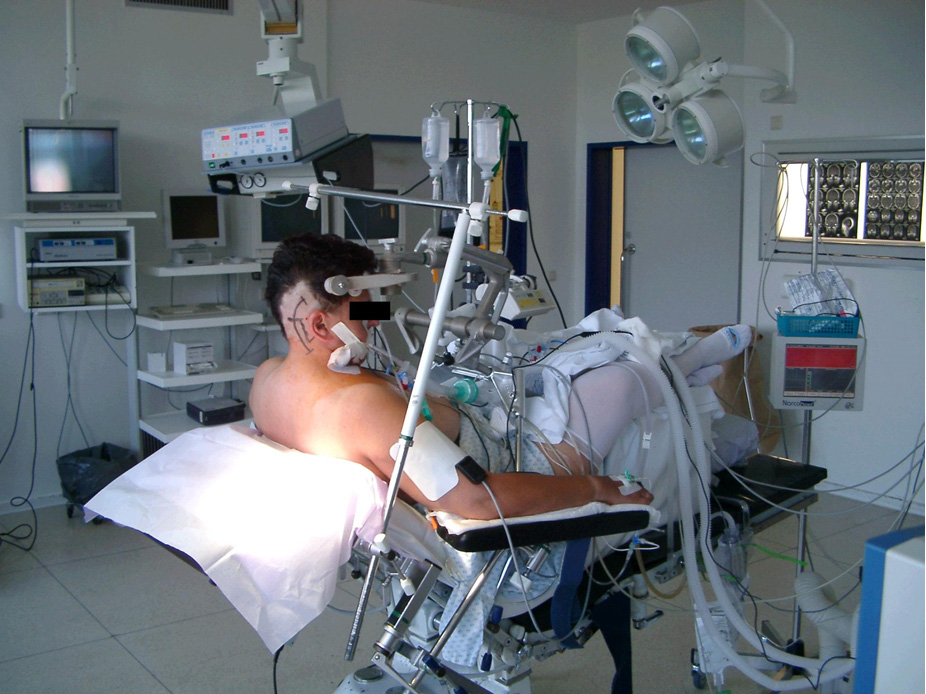
**Patient in the semi-sitting position**. The hair is shaved and the later skin incision is marked with a water resistant pencil.

We normally use a semi-sitting position for the suboccipital lateral route (Figure 23). The operation can also be carried out with the patient in a lateral position at the surgeon's discretion. After local shaving of the hair behind the pinna and disinfection of the surgical field, a curved skin incision is made. This incision crosses a line running from the asterion to the protuberantia. The nuchal muscles are split with the monopolar knife. Either osteoclastic or osteoplastic bone resection can be done. Now the sigmoid sinus and parts of the transverse sinus are exposed with the drill.

Attention should be paid to the variable emissary veins, which lead to the sigmoid sinus. First the dura should be opened under microscopic magnification in a triangular shape in the region of the pars horizontalis to gain cerebrospinal fluid and in order to relax the cerebellum.

Then the dura has to be opened near the sinus via a curved skin incision that connects with the previous dural opening.

## Conclusion

By applying the transsphenoidal, the unilateral subfrontal, pterional and lateral suboccipital approach, the surgeon can expose almost any skull base involvement. The diameters of exposure can be modified by combining different approaches. The introduction of microsurgical techniques in the 1960 s [31,32] is portrayed as a fundamental reason for the subsequently arising development in skull base surgery and the evolution of the approaches.

Complex pathologies at the skull base may need tailored approaches, which mainly have the base on one of the mentioned techniques. Extension of approaches can also be done in an interdisciplinary fashion with colleagues from other faculties like ENT or maxillofacical surgery, which is recommended by the author. While ENT can help accessing the skull base, e.g., in acoustic neuroma surgery [33] or by the help of an endoscope transnasally, maxillofacial surgery offers the wide spectrum of approaches through the oral cavity and maxilla.

## Competing interests

The authors declare that they have no competing interests.

## Authors' contributions

MS obtained clinical photos, coordinated, and drafted the manuscript. RP drafted the manuscript and participated in its coordination and design. JT, CL, AH and KB provided critical review of the manuscript for important intellectual content. All authors read and approved the final manuscript.

## Consent

A written informed consent was obtained from the patients for publication of the case reports and accompanying images. Copies of the written consents are available for review by the Editor-in-Chief of this journal.
